# Investigation of Absorption Routes of Meloxicam and Its Salt Form from Intranasal Delivery Systems

**DOI:** 10.3390/molecules23040784

**Published:** 2018-03-28

**Authors:** Csilla Bartos, Rita Ambrus, Anita Kovács, Róbert Gáspár, Anita Sztojkov-Ivanov, Árpád Márki, Tamás Janáky, Ferenc Tömösi, Gábor Kecskeméti, Piroska Szabó-Révész

**Affiliations:** 1 Institute of Pharmaceutical Technology and Regulatory Affairs, University of Szeged, Eötvös u. 6, H-6720 Szeged, Hungary; bartoscsilla@pharm.u-szeged.hu (C.B.); arita@pharm.u-szeged.hu (R.A.); anita.kovacs@pharm.u-szeged.hu (A.K.); 2 Department of Pharmacodynamics and Biopharmacy, University of Szeged, Eötvös u. 6, H-6720 Szeged, Hungary; gaspar@pharm.u-szeged.hu (R.G.); Ivanov.Anita@pharm.u-szeged.hu (A.S.-I.); marki@pharm.u-szeged.hu (Á.M.); 3 Department of Medical Chemistry, University of Szeged, Dóm tér 8, H-6720 Szeged, Hungary; janaky.tamas@med.u-szeged.hu (T.J.); tomosi.ferenc@med.u-szeged.hu (F.T.); kecskemeti.gabor@med.u-szeged.hu (G.K.)

**Keywords:** nanosized meloxicam, meloxicam potassium monohydrate, intranasal formulation, dissolution rate, in vitro diffusion, plasma drug-content, absolute bioavailability for brain

## Abstract

The aim of this article was to study the trans-epithelial absorption to reach the blood and to target the brain by axonal transport using nasal formulations with nanonized meloxicam (nano MEL spray) and its salt form known as meloxicam potassium monohydrate (MELP spray). The physicochemical properties and the mucoadhesivity of nasal formulations were controlled. In vitro and in vivo studies were carried out. These forms were first investigated in “nose-to-brain” relation. It was found that the in vitro study and in vivo study did not show any significant correlation. In vitro experiments demonstrated faster dissolution rate and higher diffusion of MELP from the spray compared with the nano MEL spray. The administration of the nano MEL spray resulted in faster absorption and constant plasma concentration of the drug after five minutes of administration as compared to MELP. The axonal transport of the drug was justified. MEL appeared in the brain tissues after the first five minutes of administration in the case of both spray forms, but its amount was too small in comparison with the total plasma concentration. The application of the nano MEL spray resulted in the same AUC in the brain as the intravenous injection. The “nose-to-blood” results predicted the nasal applicability of MEL and MELP in pain management. The “nose-to-brain” pathway requires further study.

## 1. Introduction

Besides the local effect, intranasal (IN) administration is a potential way of delivering drugs through the trans-epithelial absorption into the systemic circulation (“nose-to-blood” transport) [1,2], which results in systemic effect or, by absorption through the blood-brain-barrier (BBB), central nervous systemic (CNS) effect [3]. Some active pharmaceutical ingredients (APIs) can be delivered directly from the nasal cavity into the CNS through the axonal transport (“nose-to-brain”) [4,5,6]. A new approach is to target the brain with different API-containing drug delivery systems (DDSs) [7] in cancer (docetaxel) [8] and migraine therapy (zolmitriptan) [9] or in CNS diseases. For effective therapeutic management of schizophrenia, risperidone liposomes and paliperidone microemulsion were developed for “nose-to-brain” targeting [10,11]. Nanoparticle encapsulated tarenflurbil, piperine-loaded chitosan nanoparticles, and polyethylenimine conjugated peptide containing nose drops were formulated to deliver treatment for Alzheimer’s disease [12,13,14]. Levodopa-containing solution and powder including lactoferrin-modified rotigotine nanoparticles were administered in order to increase their therapeutic efficacy for Parkinson’s disease [15,16].

Non-steroidal anti-inflammatory drugs (NSAIDs) are mostly suggested for acute pain therapy or they are co-administered as adjuvants to enhance analgesia [17,18]. The rapid absorption of NSAIDs through the nasal mucosa into the systemic circulation and directly through the axonal route to the brain tissue can have a role in reducing pain and fever by inhibiting cyclooxygenase (COX-2) activity [19]. Few data are available on the applicability of NSAIDs in a nasal formulation. Ketorolac tromethamine-containing solution has been administered intranasally [20] and dissolved meloxicam (MEL)-containing nasal formulation was patented by Castile et al. [21]. In both cases, the main purpose was to reach the systemic circulation. MEL is a poorly water-soluble NSAID, which can be administered intranasally in order to attain an analgesic effect without a toxic effect on the nasal epithelium [22]. The solubility, the rate of dissolution, and the permeability of drugs are of great importance [23]. Our research team has experience in the field of application of different techniques for the preparation of MEL in order to produce nasal formulations with improved bioavailability. In vitro and in vivo investigations of micro- and nanonized MEL-containing IN formulations, prepared with combined wet milling, were performed, which showed better diffusion and a higher plasma AUC of the nanosized MEL-containing formulation compared to the raw or micronized drug [24]. The bioavailability of the salt form of MEL (meloxicam potassium monohydrate—MELP) [25,26] was also investigated [27]. MELP revealed a higher permeability through the synthetic membrane and a higher plasma concentration compared to the MEL-containing formulation. In every case, IN viscous formulations were prepared by adding sodium hyaluronate (HA) as a mucoadhesive agent [28], which was important from the aspect of a longer residence time. In case of these studies, the “nose-to-blood” route of drugs was investigated and the direct axonal route was not examined.

According to our previous results, the aim was the comparison study of the trans-epithelial absorption of MEL and MELP into the systemic circulation and brain by axonal transport. Nasal sprays were formulated containing nanonized MEL (nano MEL spray) and its salt form (MELP spray) by using HA as a mucoadhesive agent. The features of salt form and of the nanonized MEL enable the rapid dissolution of both materials. The formulations were controlled by physicochemical investigation and the mucoadhesivity test after the in vitro and in vivo studies were carried out.

## 2. Results and Discussion

### 2.1. PSD and Morphology

The average MEL particle size measured by laser diffraction decreased from approximately 35 µm to 0.135 µm during the 50 min milling time. The SSA of MEL rose 135-fold as a result of the nanonization. The coating effect of poly(vinyl alcohol) (PVA) prevented aggregation and the stability of the system was, therefore, improved. The particle size distribution of MELP, with D50 = 21.38 µm, fell into the range of the particle size distribution requirements of intranasally administered formulations (from 5 to 40 µm) [29].

**Figure 1:**
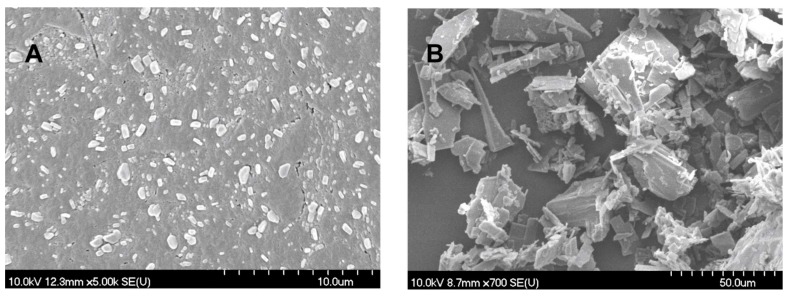
SEM images of nanonized MEL (**A**) and MELP (**B**).

The SEM images (Figure 1) provided an indication of the morphology of the particles. The nanonized MEL crystals exhibited a regular shape and a smooth surface. The treatment accounted for the smooth surfaces of the particles. The crystals of MELP were misaligned and, therefore, had a different habit (form, surface, and size).

### 2.2. Viscosity and Mucoadhesion

The mean viscosity value determined at 1001/s was 62.50 ± 2.40 mPas in the case of nano MEL spray and 71.25 ± 2.19 mPas of MELP spray. According to these results, it can be concluded that the prepared liquid formulations with low viscosity values were suitable to form intranasal sprays.

For the rheological determination of mucoadhesivity, the samples were mixed with mucin (final mucin concentration 5%) and the synergism parameter (bioadhesive viscosity component, η_b_) was calculated. The mucoadhesivity of the HA spray in the phosphate buffer (at pH 5.60), without PVA, the PVA solution without HA, the blank, and the sprays were investigated.

The synergism parameter indicated the mucoadhesivity of the samples with an interaction between the HA and the mucin (see Table 1). PVA did not reveal mucoadhesive features despite the mucin-HA interactions. Accordingly, the mucoadhesivity of blank decreased compared to the HA solution without PVA. The highest synergism was observed between the nasal spray containing nanonized MEL and the mucin. The mucoadhesivity increased 4-fold compared to the MEL-free blank and, therefore, the longest residence time could be reached. This could be explained, on the one hand, by the particle size of MEL. The nano-sized particles have increased adhesiveness to surfaces [30]. On the other hand, nano-sized MEL is in size of polymeric molecules such as HA, PVA and mucin chains, which can result in a well-structured complex and better interaction among the components and thus it shows more remarkable mucoadhesivity [24]. Adding the ionic drug (MELP) decreased the synergism, which can be explained by the interaction of the salt and the HA polymers [27,31].

### 2.3. Dissolution Testing

**Figure 2:**
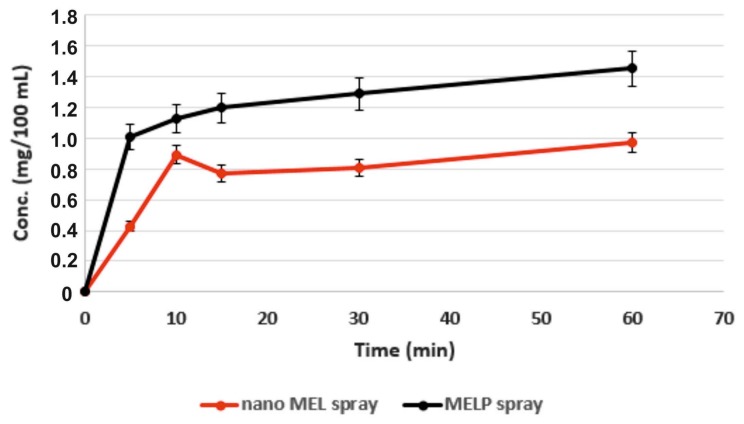
Extent of dissolution of MEL and MELP from nasal formulations at pH 5.60.

MELP showed significantly higher solubility in water (due to alkali-hydrolyzation) than MEL. However, at the pH of the nasal mucosa (pH 5.60) there were no differences, the solubility was 0.017 mg/mL in case of both materials. The in vitro dissolution test was achieved at a pH of 5.60, which simulated the conditions of the nasal cavity. Despite the same equilibrium solubilities, the difference in their rates of dissolution was perceptible. Quicker dissolution of the salt form was determined. Figure 2 shows the amount of MELP dissolved during 1 h (0.015 mg/mL) in accordance with its equilibrium solubility at the same pH. Nano MEL spray had an initial dissolution burst due to the larger surface of particles (44.8 m^2^/g). However, in this case, a lower amount of drug dissolved when compared with MELP spray.

### 2.4. In Vitro Diffusion

**Figure 3:**
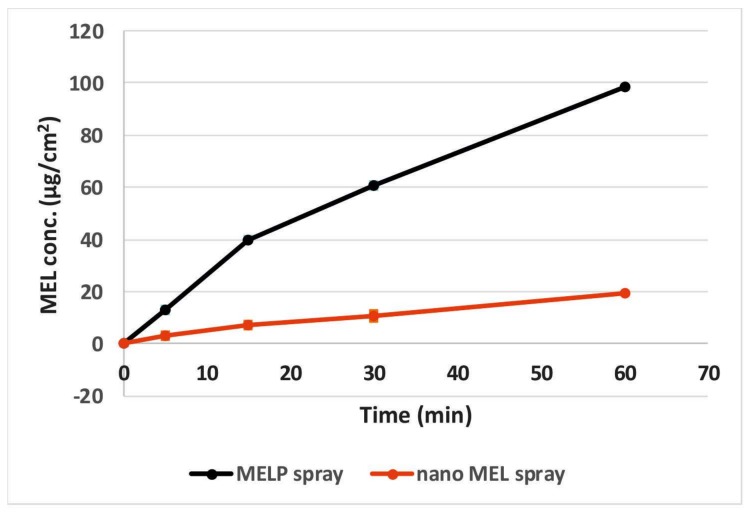
In vitro permeability of the sprays through a synthetic membrane.

Application of Side-Bi-Side^TM^ model provided the continuous stirring of the donor phase because of the homogeneous distribution of the suspended drugs. Figure 3 shows that, in spite of the same solubilities of MEL and MELP at pH 5.60, the drug from MELP spray permeated at a higher rate of pH 5.60 through the membrane than MEL from nano MEL spray, which demonstrated a faster diffusion and a higher drug concentration. This could be explained by the formation of a well-structured structure between nanonized MEL particles with PVA and HA, which resulted in a low dissolution rate of the drug.

### 2.5. In Vivo Studies

**Figure 4:**
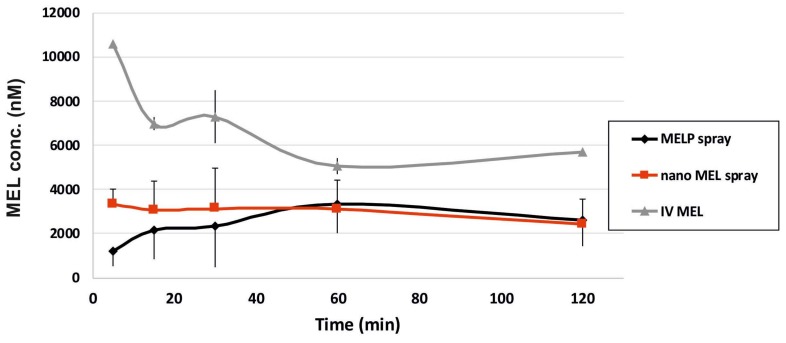
Plasma drug concentration vs. time profiles in rats after IV MEL and intranasal administration of nano MEL and MELP sprays.

Nanonized MEL and MELP containing IN spray forms were investigated through in vivo studies. Changes of drug concentration in the blood plasma as a function of time after the nasal administration of the sprays and, as a control, of the IV injection are shown in Figure 4. The plasma concentration of MEL was significantly higher in the IV group in the first 60 min. The highest measured plasma concentrations (10,554 ± 0.147 nM) were reached five minutes after the initiation of the IV injection. There were significant differences between the plasma concentrations in the case of spray forms only in the first five minutes after application. The plasma concentration increased slowly during the first approximately 60 min in the case of the MELP-containing form in contrast with nano MEL spray where peak plasma concentration was reached in the first five minutes after administration. The nanosized MEL is in the size of polymeric molecules, such as HA, PVA, and mucin chains, which can result in a well-structured complex and better interaction among the components. Therefore, it shows more remarkable mucoadhesivity and a longer residence time [24]. In both cases, a controlled release of drugs was observed one hour after the administration of IN spray formulations.

**Figure 5:**
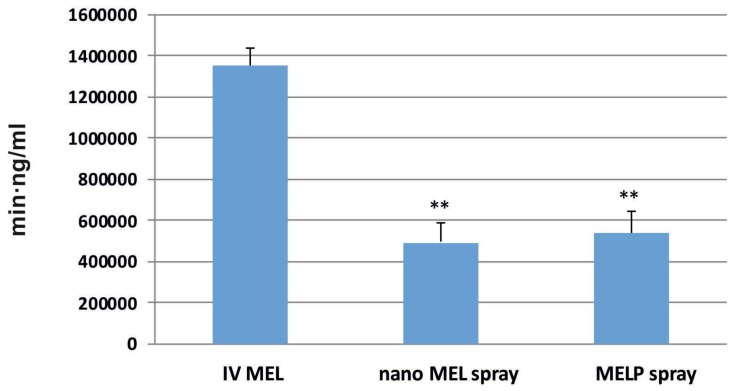
AUC values in the blood plasma of IV MEL and sprays contain nano MEL and MELP.

The AUC is proportional to the amount of drug absorbed during the investigated time interval. Plasma AUC values of IN sprays were almost the same (nano MEL spray: 492,007.3 ± 95,176.23 min·ng/mL; MELP spray: 538,393 ± 105,892.4 min·ng/mL) and significantly lower (**; p ≤ 0.01) than in the case of IV administration (1,351,000 ± 82,000 min·ng/mL) (see Figure 5). This phenomenon could be explained with the 100% bioavailability of drugs after IV administration, which is always lower in case of extravascular administration routes.

**Figure 6:**
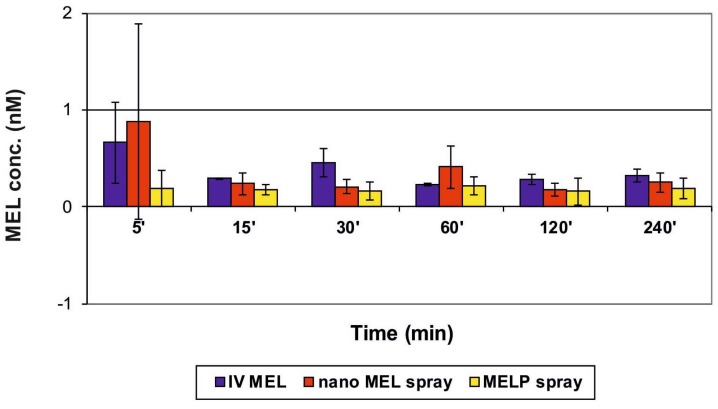
Drug concentration in the brain vs. time profiles in rats after IV MEL and intranasal administration of nano MEL and MELP sprays.

The concentration values of MEL in the brain samples vs. time profiles in case of IV and IN administration are shown in Figure 6. The application of the nano MEL containing sample resulted in a higher drug concentration (0.88 nM) in the brain tissues compared to the IV MEL and MELP. The axonal (direct “nose-to-brain”) transport of the drug was presumable by both IN spray formulations because the drug appeared in the brain in the first five minutes after administration. This could not be reached by the absorption from the nasal mucosa to the systemic circulation, which passed through BBB. One hour later, the drug-level in the brain was increased, which could already be explained with the drug absorption through the BBB from the blood plasma. The swallowing of the nasal preparation was possibly not significant since the t_max_ value was much higher (110.6 min) after the oral administration of dissolved MEL-containing viscous formulation [22].

**Figure 7:**
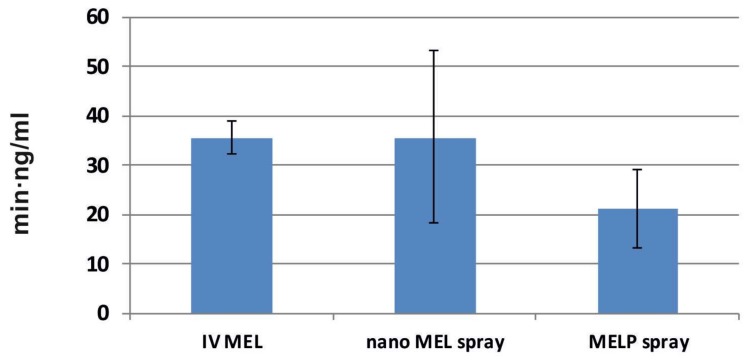
AUC values in the brain tissues of IV MEL and sprays contain nano MEL and MELP.

The calculated cerebral AUC values of the formulations did not show significant differences (see Figure 7). Administration of nano MEL particles resulted in the same AUC value (35.72 ± 8.792 min·ng/mL) as the IV administration (35.72 ± 2.355 min·ng/mL). Due to the quick dissolution of nanoparticles, a high amount of MEL could reach the brain directly by axonal transport, which resulted in the same AUC as compared with that of the IV injection where MEL should be absorbed through the BBB to target the brain.

To determine the utilization of the drug in the brain tissue, the absolute bioavailability was calculated where the brain AUC—resulted by IV MEL—was considered 100% (see Table 2). In case of nano MEL spray, the absolute bioavailability of MEL was 100% while, in case of MELP spray, it was only about 60%. These results were added from the double transport in contrast to the IV MEL.

The cerebral drug targeting efficiency index (% DTE) reflects the relative accumulation of the drug in the brain following IN administration as compared to systemic (IV) administration. In case of IN formulations, % DTE data were above 100% in both cases. Therefore, drug delivery to the brain following IN administration was more efficient when compared to systemic administration. This could be explained with both axonal and epithelial routes of drugs compared to IV administration where the APIs could reach the brain tissues only through the BBB.

## 3. Experimental Set-Up

### 3.1. Materials

MEL (4-hydroxy-2-methyl-*N*-(5-methyl-2-thiazolyl)-2H-benzothiazine-3-carboxamide-1,1-dioxide) as a water-insoluble (0.040 mg/mL at 37 °C) agent and MELP (4-hydroxy-2-methyl-*N*-(5-methyl-2-thiazolyl)-2H-benzothiazine-3-carboxamide-1,1-dioxide potassium monohydrate) as a water-soluble (13.10 mg/mL at 37 °C) drug were obtained from EGIS Ltd. (Budapest, Hungary). Piroxicam, the internal standard for the HPLC method, was purchased from the Alfa Aeasar Co. (Alfa Aeasar GmbH & Co. KG, Karlsruhe, Germany). The grinding additive, PVA 4-98 (Mw ~27,000), was procured from Sigma Aldrich (Sigma Aldrich Co. LLC, St. Louis, MO, USA). HA (Mw = 1400 kDa) was obtained as a gift from Gedeon Richter Plc. (Budapest, Hungary). Mucin (porcine gastric mucin type II) was obtained from Sigma Aldrich (Sigma Aldrich Co. LLC, St. Louis, MO, USA).

### 3.2. Methods

#### 3.2.1. Sample Preparation

The content of intranasal spray forms are presented in Table 3. For the preparation of nano MEL spray, combined wet milling techniques (a combination of planetary ball and pearl milling) was applied to prepare the pre-dispersion. During milling, PVA was used as a coating polymer, which promoted the separation of particles from each other. 0.5 g PVA was dissolved in 17.5 mL phosphate buffer (PBS) (at a pH of 5.60, the pH of the nasal mucosa) to prepare a dispersant medium in which 2.0 g of MEL was suspended. The suspension was wet-milled in the planetary ball mill (Retsch PM 100) (Retsch GmbH, Haan, Germany) for 50 min using zirconium dioxide beads, which were 0.3 mm in diameter in order to reach the nano-range. The milling medium for this study was zirconium dioxide beads. The IN formulation (nano MEL spray) was prepared directly from the pre-dispersion by diluting with phosphate buffer (pH 5.60) in order to reach 2 mg/mL concentration of MEL and 0.15 g HA as a natural anionic polysaccharide was added. Therefore, the final formulation contained 5 mg/mL HA.

MELP spray was developed with 2 mg/mL MELP and 5 mg/mL HA using phosphate buffer as dispersant medium.

Intravenous (IV) injection (IV MEL) was prepared by the dilution of passable injection with a concentration of 15 mg/1.5 mL (Meloxicam-Zentiva, Prague, Czech Republic) to reach the final concentration (0.15 mg/mL). The components of injection were meglumine, poloxamer 188, glycine, sodium hydroxide (for pH adjustment), sodium chloride, glycopherol, and water for injection.

#### 3.2.2. Determination of Particle Size Distribution (PSD)

The volume-based PSD of MEL in the pre-dispersions was measured by using laser diffraction (Mastersizer 2000) (Malvern Instruments Ltd., Worcestershire, UK) with the following parameters: 300RF lens, small volume dispersion unit (2500 rpm), refractive index for dispersed particles 1.720, and refractive index for dispersion medium 1.330. A dynamic laser light scattering method was used to determine the PSD. Water was applied as a dispersant and the obscuration was in the range of 11–16% for all measurements. In all cases, the volume-weighted PSDs, D10, D50, and D90 (where, for example, D50 is the maximum particle diameter below which 50% of the sample volume exists—also known as the median particle size by volume) were determined and evaluated. The size analysis was repeated three times. The specific surface area (SSA) was derived from the PSD data. The assumption was made that all of the particles measured were spherical.

#### 3.2.3. Image Analysis (SEM)

Pre-dispersions were dried in a vacuum dryer (Binder GmbH, Tuttlingen, Germany) at 40 °C in order to obtain solid products for physical-chemical investigations. After drying, the shape and surface characteristics of the samples were visualized by using SEM (Hitachi S4700, Hitachi Scientific Ltd., Tokyo, Japan). The samples were coated with gold–palladium under an argon atmosphere by using a gold sputter module in a high-vacuum evaporator and the samples were examined at 15 kV and 10 μA. The air pressure was 1.3–13 MPa.

#### 3.2.4. Rheology and Muco Adhesion

Rheological measurements were taken at 37 °C with a Physica MCR101 rheometer (Anton Paar GmbH, Graz, Austria). A concentric cylindrical measuring device with a diameter of 10.835 mm was used for the experiment. Viscosity curves were plotted to determine the viscosity of the samples. In the shear rate interval from 0.1 to 1001/s, viscosity values were plotted.

To clarify the roles of nano MEL and MELP in muco-adhesion, samples were prepared with and without mucin. The samples containing mucin were stirred for 3 h before the measurements (the final mucin concentration was 5% *w*/*w*). The mucoadhesivity was determined on the basis of the rheological synergism between the polymer and the mucin. The synergism parameter (bioadhesive viscosity component, η_b_) can be calculated from the following equation.

η_b_ = η_t_ − η_m_ − η_p_

where η_t_ is the viscosity of the mucin and polymer-containing samples and η_m_ and η_p_ are the viscosities of the mucin and nasal spray, respectively [32]. Three parallel measurements were used to determine the viscosity values (η_t_, η_m_, and η_p_) and the standard deviations.

#### 3.2.5. In Vitro Dissolution Test

The paddle method (USP dissolution apparatus, type II; Pharma Test, Hainburg, Germany) was used to examine the rates of dissolution of MEL- and MELP-containing intranasal spray forms and to determine the drug release profile from the samples. The test was carried out under nasal conditions. The medium was 100 mL PBS of pH 5.60 in which 3 mL of the samples were used. The paddle was rotated at 50 rpm and the sampling was performed for 60 min. After filtration, the drug contents of the aliquots were determined using spectrophotometry (Unicam UV/VIS Spectrophotometer) at 364 nm.

#### 3.2.6. In Vitro Diffusion Study

In vitro permeability studies were carried out on a modified horizontal Side-Bi-Side™ cell (Grown Glass, New York, NY, USA), which modeled the conditions of nasal cavity. The validation of the in vitro investigations were accomplished by comparing the results and measuring on the Side-Bi-Side™ cell model with the results of the Franz-diffusion cell system (Hanson Microette Topical and Transdermal Diffusion Cell System) (Hanson Research, Chatsworth, CA, USA) [24]. The intranasal spray was used as a donor phase, which was prepared using phosphate buffer (PBS) of pH 5.60. This represents the pH of the nasal cavity. The synthetic membrane (PALL Metricel membrane), impregnated with isopropyl myristate, between the donor and acceptor compartments simulated the lipophilic nasal mucosa. The pH (7.4) of the acceptor phase corresponded with the pH of the blood. The volumes of the donor and the acceptor phase were the same (3.0 mL) and had a 0.69 cm^2^ diffusion area. The temperature of the phases was 37 °C (Thermo Haake C10-P5, Sigma, Aldrich Co.) and the rotation rate of the stir-bars was set to 100 rpm. Both phases were stirred with a magnetic stirrer, which simulated the movements of the cilia and the blood circulation. Aliquots (2.0 mL) were taken from the acceptor phase by pipette and were replaced with fresh receiving medium at 5, 10, 15, and 60 min of the measurement. The amount of MEL or MELP diffused was determined spectrophotometrically (Unicam UV/VIS) at 364 nm. Each sample was measured three times.

#### 3.2.7. In Vivo Studies

##### IN Administration, Blood Sample Collection, and Brain Removal

The IN formulations contained 2 mg/mL MEL and 5 mg/mL HA. A dose of 60 μg MEL per animal was administered into the right nostril of 160–180 g male Sprague–Dawley rats (*n* = 4) via a pipette. The administration was carried out under isoflurane short anesthesia. As a control, iv delivery of the injection containing MEL in solution form (IV MEL) was given at a dose of 60 μg MEL per animal to rats (*n* = 4). At predetermined time points (5, 10, 15, 30, 45, 60, 90, 120, 180, and 240 min) after MEL dosing, the blood of the rats—under deep isoflurane anesthesia—was collected into heparinized tubes by cardiac puncture. Then the animals were sacrificed by decapitation and brain tissues were quickly removed, rinsed in ice-cold PBS, divided into left and right hemispheres, weighed, and stored at −80 °C until assayed. The experiments were performed according to the EU Directive 2010/63/EU for animal experiments and were approved by the Hungarian Ethical Committee for Animal Research (permission number: IV/1247/2017).

##### Sample Preparation of Rat Plasma and Brain

Plasma samples were centrifuged at 1500 *g* for 10 min at 5 °C. Separated plasma samples were stored at −80 °C until it was time for extraction and analysis. To a 90 µL of plasma sample, 10 µL 0.1% aqueous formic acid and 300 µL acetonitrile containing piroxicam (internal standard at 12.5 ng/mL concentration) were added and the mixture was spun for 60 s. The mixture was allowed to rest for 30 min at −20 °C to support protein precipitation. Supernatant was obtained by the centrifugation of the mixture for 10 min at 10,000 *g* at 4 °C. 20 µL of clear supernatant was diluted by 380 µL 0.1% aqueous formic acid and spun for 30 s. Finally, 5 µL was injected into the LC–MS/MS system for analysis. The rat plasma calibration standards of MEL were prepared by moving the working standard solutions (1–1000 ng/mL) into a pool of drug-free rat plasma. The procedure described above was followed. Calibration standards consisted of 90 µL pooled plasma samples, 10 µL MEL standard solution (in 0.1% aqueous formic acid), and 300 µL acetonitrile containing piroxicam (internal standard at 12.5 ng/mL concentration). Solutions containing 6.25 ng/mL and 25 ng/mL MEL were used as QC samples. 20 µL supernatant was taken out from both of the calibration standards and QC samples, diluted with 380 µL 0.1% aqueous formic acid, and 5 µL was analyzed by LC–MS/MS.

The right hemispheres (0.8–1 g) of rats were homogenized in 5 mL 1% aqueous acetic acid using an Ultra-Turrax homogenizer in an ice bath for 2 × 30 s interrupted by 30 s cooling. To 100 µL of brain homogenate, 20 µL 0.1% aqueous formic acid and 20 µL piroxicam internal standard (1.5625 ng/mL in 0.1% aqueous formic acid) were added and the mixture was vortex-mixed for 60 s. After centrifugation for 10 min at 10,000 *g* at 4 °C, 40 µL supernatant was injected into the LC–MS/MS system for analysis.

Rat brain calibration standards of MEL were prepared by adding the working standard solutions (1–1000 ng/mL) to a pool of drug-free rat brain. The procedure described above was followed. Calibration standards consisted of 100 µL pooled brain samples, 20 µL MEL standard solution (in 0.1% aqueous formic acid), and 20 µL piroxicam internal standard (1.5625 ng/mL in 0.1% aqueous formic acid). Solutions containing 6.25 ng/mL and 25 ng/mL MEL were used as QC samples. After centrifugation, 40 µL was analyzed by LC–MS/MS.

##### LC–MS/MS Analysis of MEL

The quantitative analysis of MEL was performed after chromatographically separating by using mass spectrometry. An Agilent Liquid Chromatography System series 1100 including Micro Vacuum Degasser, Capillary Pump, µ-WPS autosampler (Agilent Technologies, Waldbronn, Germany) connected to a Q Exactive^TM^ Plus Orbitrap mass spectrometer (Thermo Fisher Scientific, San Jose, CA, USA) equipped with a heated ESI ion source (HESI) was applied to the analysis. Gradient chromatographic separation was performed at room temperature on a Luna C8(2) Mercury column (20 mm × 2.0 mm, particle size 5.0 µm) protected by a C8 guard column (2 × 2 mm) (Phenomenex, Torrance, CA, USA) by using ammonium formate (15 mM, pH = 3) as Solvent A and acetonitrile as Solvent B (see Table 4). The calibration curve was shown to be linear over the concentration range of 1–1000 ng/mL.

The mass spectrometer was used in a positive mode with the following parameters of the H-ESI source: spray voltage at 3.5 kV, capillary temperature at 253 °C, aux gas heater temperature at 406 °C, sheath gas flow rate at 46 L/h, aux gas flow rate at 11 L/h, sweep gas flow rate at 2 L/h, S-lens RF level at 50.0 (source auto-defaults). Parallel-reaction-monitoring (PRM) mode was used for quantifying by monitoring the transitions: m/z 352 → 115 and 352 → 141 for MEL and m/z 332 → 95 and 332 → 121 for piroxicam. The normalized collision energies (NCE) for specific quantification were optimized to maximize sensitivity. The NCE was 24 for MEL and 29 for piroxicam. A valve placed after the analytical column was programmed to switch flow onto MS only when analytes of interest elute from the column (brain samples: 0.9–2.1 min; plasma samples: 0.7–2.0 min) to prevent excessive contamination of the ion source and ion optics. Washing procedures of the autosampler before and after injecting samples were programmed in order to avoid carry-over of analytes.

Data acquisition and processing were carried out using Xcalibur and Quan Browser (version 4.0.27.19) software (Thermo Fisher Scientific, San Jose, CA, USA).

##### Calculations of the Area under the Time-Concentration Curve (AUC) and Statistical Analysis

The area under the curve (AUC) of the time (min)–concentration (ng/mL) curves of each animal and the statistical analysis were performed with Prism 5.0 software (GraphPad Software Inc., La Jolla, CA, USA). All reported data are means ± SD. The unpaired *t*-test was used to determine statistical significance. Changes were considered statistically significant at *p* < 0.05. The ratio of AUC value, after IN application in brain in comparison with the AUC of IV administration (absolute bioavailability for brain—% abs. BA for brain) was determined according to the formula below.

$$\%\;abs.\;BA\;for\;brain\;=\;\frac{AUCbrain\;IN}{AUCbrain\;IV}\times 100.$$

Drug targeting efficiency percentage (%DTE)—relative exposure of the brain to the drug following IN administration vs. systemic administration—was calculated according to the formula below.

(1) $$\%DTE=\frac{\left(\frac{AUC_{brain}}{AUC_{blood}}\right)IN}{\left(\frac{AUC_{brain}}{AUC_{blood}}\right)IV}\times 100$$

The value of %DTE can range from −∞ to ∞, and the values higher than 100% indicate more efficient drug delivery to the brain following IN administration when compared to the systemic administration [33].

## 4. Conclusions

The results confirmed both trans-epithelial and axonal transport of MEL and MELP. Nanonized MEL showed better absorption and constant plasma concentration of the drug after five minutes of administration when compared with MELP. It is connected to the high specific surface area, the enhanced saturation solubility [34], and the high mucoadhesivity of the nano MEL spray [35]. The reduction of the particle size of MEL into the nano range resulted in the increased adhesiveness to surfaces [30], which ensured the rapid appearance of the drug in the blood plasma. Initial slow absorption of MELP could be explained with low transepithelial activity of its dissociated form, which decreased the mucoadhesivity of the spray. Plasma AUC values were similar and significantly lower in case of the administration of IN forms compared with MEL-containing IV injection. It can be stated that the “nose-to-blood” pathway is determinative in the transport of MEL. This is consistent with literary data [20,21,22] where MEL and ketorolac tromethamine were investigated as pain killers. Otherwise, the ketorolac tromethamine containing nasal spray was the first on the market for pain relief.

The axonal transport of MEL was assumed when the drug appeared in the brain tissues in the first five minutes after the application of nasal sprays. In terms of cerebral AUC values, the absolute bioavailability of nano MEL spray was 100% when compared with the IV injection. This can also be related to the advantageous properties of the nanonized MEL. The IN administration of MEL and MELP resulted in higher relative accumulation of the drugs (% DTE) in the brain by the axonal (“nose-to-brain”) and BBB (“blood-to-brain”) transport when compared with IV administration. It is also important to consider the axonal transport and the pathway through the BBB. Additionally, the absorbed amount of MEL is less than 0.001% of the total plasma concentration. Further development and experiments with a higher dose of drugs are necessary to enhance the directly absorbed amount of MEL into the brain. Few data points are available on IN application of NSAIDs with the aim of preventing Alzheimer’s disease. This can be related to their anti-inflammatory effect, which is a new approach to target the brain with NSAIDs [36,37].

## Figures and Tables

**Table 1:** Calculated synergism parameters at shear rate of 1001/s of spray forms.

	HA Spray	PVA Solution	Blank (HA + PVA)	Nano MEL Spray	MELP Spray
Synergism parameters (mPa*s)	165 ± 30	−10 ± 1	90 ± 30	355 ± 25	59.4 ± 20

**Table 2:** Calculated parameters of spray forms apply IV administration as a benchmark.

	abs. BA for Brain (%)	AUC_brain_/AUC_blood_	DTE (%)
IV MEL	100	2.644 × 10^−5^	100
nano MEL spray	100	7.26 × 10^−5^	274.58
MELP spray	59	3.93 × 10^−5^	148.64

**Table 3:** Content of intranasal spray formulations.

	MEL (mg)	MELP (mg)	PVA (mg)	HA (mg)	PBS of pH 5.60 (mL)
nano MEL spray	60.0	-	15.0	150.0	ad 30.0
MELP spray	-	60.0	-	150.0	ad 30.0

**Table 4:** The gradient applied for analysis.

t (min)	%B	Flow Rate (µL/min)
0	40	250
0.5	40	250
2	70	250
2.1	90	600
2.5	90	600
2.6	40	600
4.0	40	600
4.1	40	250
4.5	40	250
